# Nuclear entry of DNA viruses

**DOI:** 10.3389/fmicb.2015.00467

**Published:** 2015-05-13

**Authors:** Nikta Fay, Nelly Panté

**Affiliations:** Department of Zoology, University of British ColumbiaVancouver, BC, Canada

**Keywords:** Nuclear import, nuclear envelope, nuclear pore complex, nucleoporins, virus nuclear entry, virus nuclear import, DNA virus

## Abstract

DNA viruses undertake their replication within the cell nucleus, and therefore they must first deliver their genome into the nucleus of their host cells. Thus, trafficking across the nuclear envelope is at the basis of DNA virus infections. Nuclear transport of molecules with diameters up to 39 nm is a tightly regulated process that occurs through the nuclear pore complex (NPC). Due to the enormous diversity of virus size and structure, each virus has developed its own strategy for entering the nucleus of their host cells, with no two strategies alike. For example, baculoviruses target their DNA-containing capsid to the NPC and subsequently enter the nucleus intact, while the hepatitis B virus capsid crosses the NPC but disassembles at the nuclear side of the NPC. For other viruses such as herpes simplex virus and adenovirus, although both dock at the NPC, they have each developed a distinct mechanism for the subsequent delivery of their genome into the nucleus. Remarkably, other DNA viruses, such as parvoviruses and human papillomaviruses, access the nucleus through an NPC-independent mechanism. This review discusses our current understanding of the mechanisms used by DNA viruses to deliver their genome into the nucleus, and further presents the experimental evidence for such mechanisms.

## Introduction

Viruses are opportunistic pathogens that infect a host by attacking their cells and hijacking the cellular machinery to replicate, produce progeny virus particles, and spread infection. A few RNA viruses and almost all DNA viruses replicate themselves in the nucleus of their host cells. To accomplish this, their viral genome must enter the host nucleus. Entry into the nucleus is also achieved by many viral proteins (reviewed by Fulcher and Jans, 2011), which either assist in viral replication or are required for the formation of progeny viral subparticles or capsids. Nuclear export is also at the basis of viral infections because many viral pathogens assemble in the nucleus and must then exit the nucleus. Thus, viral hijacking of nuclear transport is important for several viruses to complete their infection cycle.

Physiological transport of macromolecules between the cytoplasm and the nucleus is highly selective and occurs through nuclear pore complexes (NPCs), large protein assemblies embedded in the nuclear envelope (NE). The NPC allows passive diffusion of ions and small molecules through aqueous channels with a diameter of about 9 nm. Nuclear import or export of molecules larger than this diffusion limit, and up to 39 nm in diameter (Pante and Kann, 2002), is a highly selective process that requires a signal residing on the transported molecule and soluble import or export receptors that recognize these signals (Wente and Rout, 2010). Few years ago it was largely assumed that viruses or viral capsids were either small enough to enter the nucleus through the NPC or disassemble in the cytoplasm releasing subviral particles that then enter the NPC. However, recent progress in the characterization of the nuclear import of several viruses has drastically changed the perception of how viruses use the cellular machinery for nuclear import. The emerging picture is that each virus has evolved a unique strategy to deliver its genome into the nucleus. These strategies include the use of all or some of the components of the cellular nuclear transport machinery, but also unexpected mechanisms such as the use of the NPC for capsid disassembly or viral nuclear entry through the nuclear membranes instead of the NPC.

Retroviruses, such as the human immunodeficiency virus type 1, and orthomyxoviruses, such as influenza A virus, are RNA viruses that also deliver their genomes into the nucleus of the cells they infect. Since these viruses are significant human pathogens, their mechanisms of nuclear transport have been the focus of intense research, and several excellent reviews describing their nuclear transport have been published in recent years (Boulo et al., 2007; Di Nunzio, 2013; Hutchinson and Fodor, 2013; Matreyek and Engelman, 2013; Eisfeld et al., 2015). Thus, we do not cover nuclear import of RNA viruses in this review, and instead present the current knowledge and recent advancements in studies of the nuclear entry mechanisms used by DNA viruses. We first briefly summarize the principles of cellular nuclear transport, discuss the experimental approaches used to study viral nuclear import, and then discuss the nuclear entry strategies used by several DNA viruses, including hepatitis B virus (HBV), herpes simplex virus 1 (HSV1), baculoviruses, adenoviruses, parvoviruses, the polyomavirus simian virus 40 (SV40), and human papillomavirus (HPV). The key features of these viruses are summarized in **Table 1**.

**Table 1:** Summary of key features of the DNA viruses discussed in this review.

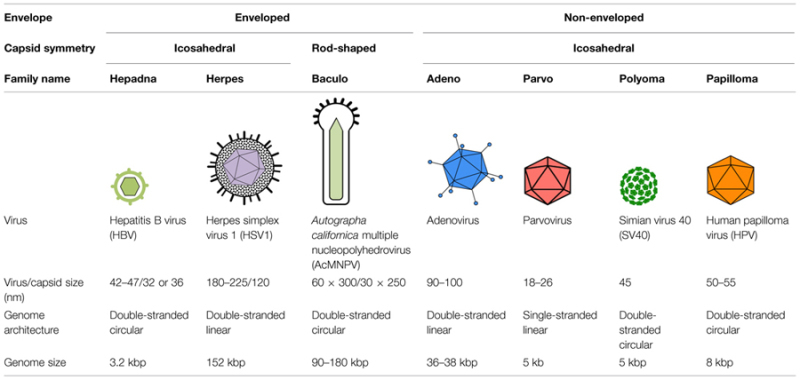

## Overview of the Cellular Nuclear Transport Machinery

Although several macromolecules enter the nucleus (e.g., spliceosomal and 5S ribosomal ribonucleoprotein complexes, and mitochondrial DNA), the nuclear import of proteins is the best-characterized process. Key players involved in this process are: the NPC, nuclear localization sequences (NLSs) on the transported proteins, nuclear transport receptors (NTRs) that recognize the NLSs in the transported proteins, and the small Ras-like GTPase Ran (reviewed by Wente and Rout, 2010).

**FIGURE 1:**
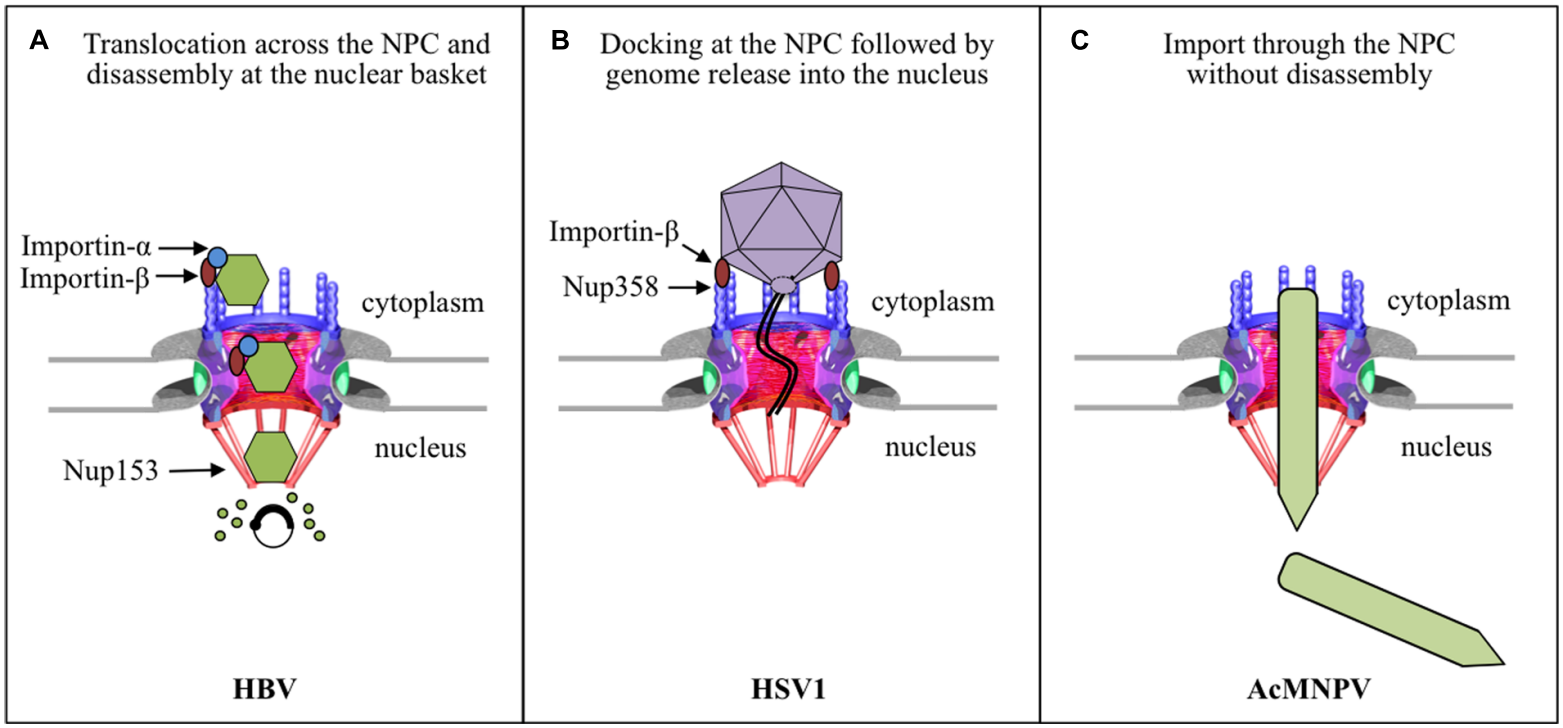
**Strategies used by enveloped viruses for nuclear entry of their genomes. (A)** HBV capsids bind and cross the NPC in an importin-α and -β dependent manner. The capsid then binds directly to Nup153 at the nuclear basket, which somehow triggers disassembly of the capsid and nuclear entry of the viral genome. **(B)** The HSV1 capsid docks with a distinct orientation to the NPC cytoplasmic filament by binding to Nup358 in an importin-β and Ran dependent manner. The genome is delivered into the nucleus through the NPC by a unique mechanism of genome release through the capsid portal, and the empty capsid remains at the cytoplasmic side of the NPC. **(C)** The intact nucleocapsid of the baculovirus AcMNPV enters the nucleus through the NPC, but the roles of any viral or cellular components remain to be determined.

The NPC is a large proteinaceous structure (125 MDa in vertebrates; Reichelt et al., 1990), made of only 30 different proteins called nucleoporins (Nups). These are present in multiple copies and are arranged in a structure of 120 nm in diameter and 70 nm in height. The NPC has eightfold rotational symmetry, and structurally is described as having three rings: a central framework ring embedded in the NE, which is sandwiched between a cytoplasmic and a nuclear ring. Extending from the cytoplasmic ring are eight cytoplasmic filaments, and attached to the nuclear ring is a basket-like structure, termed the nuclear basket (see NPC diagram in **Figure 1**). The central framework ring contains a large central channel of about 50 nm in diameter through which molecules enter or exit the nucleus. Nups contain hydrophobic phenylalanine glycine (FG) sequence repeat motifs that line this channel. NTRs bind to these FG-Nups to transiently melt the barrier at the center of the NPC, thus allowing nuclear transport (reviewed by Wente and Rout, 2010).

Proteins destined to enter the nucleus bear a biochemical “pass code” – one or more short stretches of specific amino acid sequences termed NLSs (Lange et al., 2010; Marfori et al., 2011). There are many types of NLSs and each one is recognized by a specific NTR – with the exception of the NTR importin-β, which recognizes the classical NLS through an adaptor protein, importin-α. The NTRs belong to a superfamily of proteins termed the karyopherin-β-family; there are over 20 β-karyopherins in human cells (Chook and Suel, 2011). The GTPase Ran regulates the association and dissociation of the NTR with the NLS-containing cargo; NTR binds to NLS-cargo in the presence of Ran-GDP, and dissociates from it in the presence of RanGTP (Gorlich et al., 1996; Izaurralde et al., 1997; Fried and Kutay, 2003).

In contrast to nuclear import of proteins, less is know about nuclear import of DNA, even though studies on nuclear transport of DNA have already been published decades ago (Capecchi, 1980). Nevertheless, two recent studies addressed the role of NTR in the nuclear import of exogenous and endogenous DNA. The first found that nuclear import of exogenous DNA is mediated by the NTR transportin-1, with histones acting as an adaptor between tranportin-1 and the DNA (Lachish-Zalait et al., 2009). The second study, reported that the NTR importin-7 mediates nuclear import of mitochondrial DNA (Dhanoya et al., 2013), which is transported from mitochondria to the nucleus in eukaryotic cells. It is not clear whether importin-7 binds directly to the DNA or uses an adaptor protein. Since some DNA viruses transport their genome into the nucleus, studies on nuclear import of DNA viruses will help unveil the cellular mechanism for endogenous DNA.

## Experimental Approaches to Study Viral Nuclear Import

Two well-established systems of studying nuclear import have provided much of our insight into its mechanisms, and these systems have been adapted for the study of nuclear import of viruses. These are the *Xenopus* oocyte nuclear import assay and the *in vitro* nuclear import assay with digitonin-permeabilized cells. In the oocyte system, the substrate to be studied (protein, virus, or viral capsid) is introduced by microinjection into the cytosol of the oocyte, and nuclear import is monitored by electron microscopy (EM; Pante, 2006; Au et al., 2010). In the digitonin-permeabilized cell system, the plasma membrane but not the NE becomes permeabilized with the detergent digitonin (Adam et al., 1990; Cassany and Gerace, 2009), which preferentially extracts cholesterol present in the plasma membrane but not in the nuclear membranes. This treatment and subsequent washing releases soluble cytosolic components; thus for this assay the permeabilized cells are incubated with the fluorescently labeled substrate, exogenous cytosol or purified recombinant NTRs and Ran, and an energy-regenerating system. Nuclear import is then monitored by fluorescence microscopy or indirect immunofluorescence microscopy if the import cargo is not fluorescently labeled. Other systems used for the study of nuclear import of viruses are microinjection of tissue culture cells and *in vitro* nuclear import assays with purified nuclei. However, because viruses are often modified during cellular entry, results obtained using microinjection or *in vitro* nuclear import assays are then validated with experiments in infected cells.

To study the involvement of the NPC in viral nuclear import a common experimental strategy is the use of the lectin wheat germ agglutinin (WGA), which binds to the *N*-acetylglucosamine residues found on Nups (Finlay et al., 1987; Dabauvalle et al., 1988), or anti-Nup antibodies. Both treatments block the NPC and inhibit nuclear transport. Antibodies against a particular Nup or depletion of a particular Nup by RNA interference (RNAi) have also been used to study the role of a particular Nup in the mechanism of nuclear import of viruses. Finally, to determine the role of viral components, virus or viral capsids carrying mutations of a particular protein are used.

## Nuclear Entry of Enveloped DNA Viruses

The first barrier viruses have to overcome to infect a host cell is the plasma membrane. Enveloped viruses often have a fusion protein that promotes fusion of the viral envelope with the plasma membrane or with the endosomal membrane after their uptake by endocytosis. This results in the release of viral capsids into the cytoplasm of the host cell. Subsequently the capsid uses the host cytoskeleton to travel to the nuclear periphery. Although enveloped viruses may share a similar cell entry mechanism due to the existence of their membranes, their nuclear entry mechanisms are very unique, most likely due to their differing features, such as size and architecture of the virus, and genome size. As summarized in **Figure 1**, we have now come to understand the nuclear entry strategy used by three enveloped viruses to deliver their genome into the nucleus.

## Hepatitis B Virus

Human HBV is an enveloped virus with a diameter of 42–47 nm, containing an icosahedral capsid with a partially double-stranded DNA genome of 3.2 kbp (Seeger et al., 2013). The capsid is composed of a single protein called the core protein (185 amino acids, 21 kDa); there are two types of capsids: one with diameters of 32 nm and containing 180 copies of the core protein, and the second with diameter of 36 nm and containing 240 copies of the core protein (Vanlandschoot et al., 2003). Studies of the early HBV infection steps have been challenging because human hepatocytes, the physiological host, are difficult to maintain in tissue culture. Nevertheless, the recent development of alternative liver-derived cell lines able to support HBV infection *in vitro*, has considerably advanced our understanding of HBV cell entry in the last few years. Thus, it has only recently been established that HBV enters cells by caveolin-1- or clathrin-mediated endocytosis depending on the cell line used (Macovei et al., 2010; Huang et al., 2012). The DNA-containing capsid is then released into the cytoplasm by the fusion of the viral envelope with endosomal membranes, and travels along the microtubule network toward the nucleus (reviewed by Kann et al., 2007). The HBV capsid then uses a unique mechanism to deliver its genome into the nucleus: the intact capsid crosses the NPC, in a phosphorylation- and importin-α/β-dependent manner, and undergoes complete disassembly at the NPC nuclear basket releasing the uncoated genome into the nucleus (**Figure 1A**).

**FIGURE 2:**
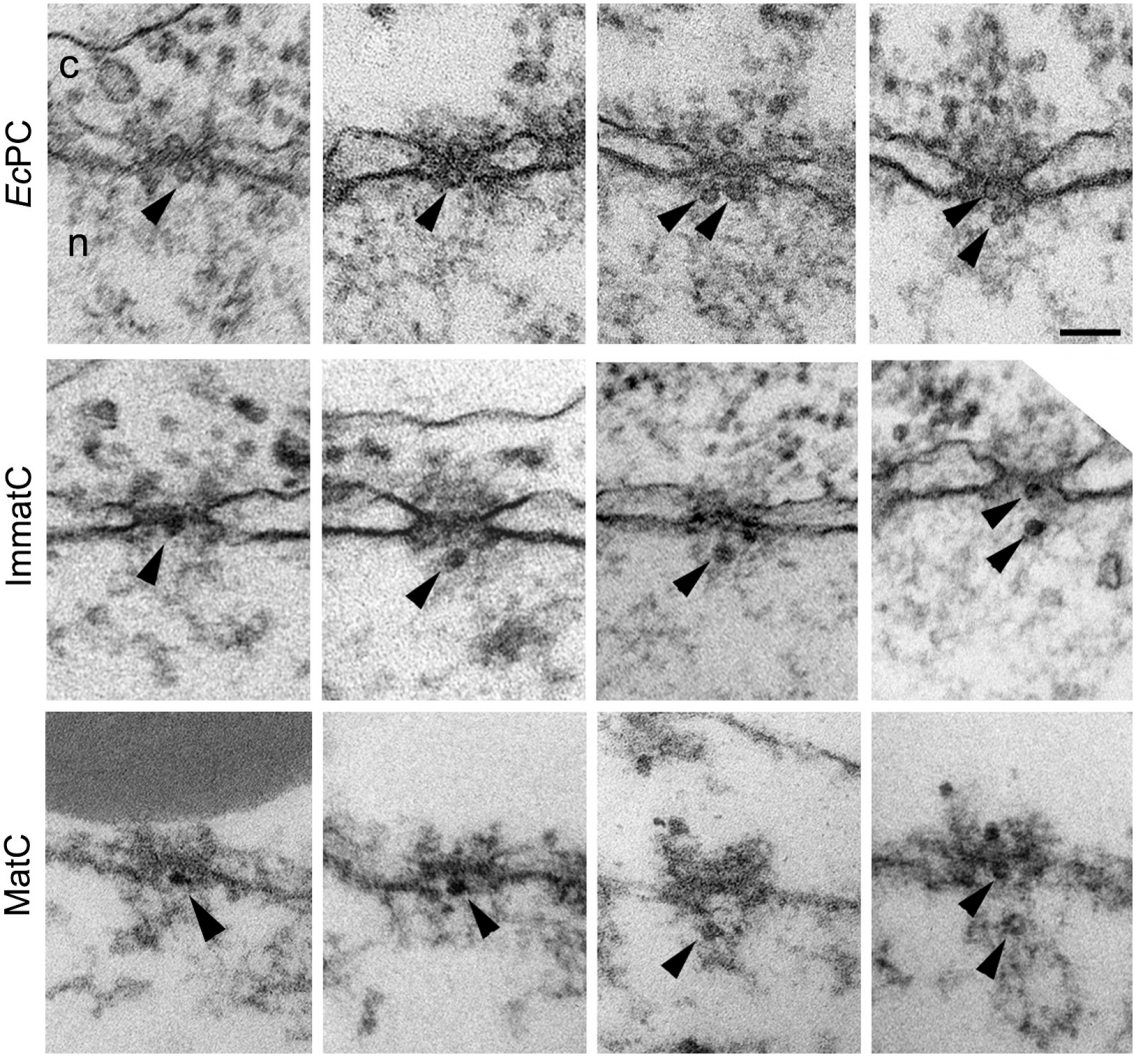
**Hepatitis B virus capsids cross the NPC intact and get arrested within the NPC nuclear basket, depending on genome maturation.** Electron micrographs of NPC cross-sections from *Xenopus* oocytes microinjected with phosphorylated recombinant HBV capsids (*Ec*PC), HBV capsids containing the mature viral genome (MatC), or HBV capsids that did not contain the mature viral genome (ImmatC). While all three types of capsids cross the NPC intact, recombinant capsids and immature capsids remain arrested within the NPC nuclear basket, while mature capsids are released into the nucleus. Scale bar, 100 nm; n, nucleus; c, cytoplasm. Arrowheads point to capsids associated with the nuclear face of the NPC. Some of the micrographs have been reproduced with permission from Rabe et al. (2003).

Due to the unavailability of an *in vitro* infection system that allows the study of the early steps in HBV infection, studies of nuclear import of HBV have used nuclear import assays with digitonin-permeabilized cells and *Xenopus* oocyte microinjection. Using the former assay and recombinant HBV capsids obtained by expressing the core protein in *Escherichia coli,* Kann et al. (1999) first demonstrated that these capsids bind to the NE of digitonin-permeabilized cells in the presence of cytosol and an energy-regenerating system, yielding a characteristic fluorescent rim staining of the nucleus only when the capsid is phosphorylated *in vitro* prior to the assay (Kann et al., 1999). The core protein has two classical NLSs at its arginine rich C-terminus (Yeh et al., 1990; Eckhardt et al., 1991; Li et al., 2010). Because the C-terminus is not exposed on the capsid (Zlotnick et al., 1997), it has been suggested that phosphorylation of the core protein exposes these NLSs, which then mediates the binding of the capsid to the NPC (Kann et al., 1999). In agreement with this, binding of the recombinant capsids to the NE of digitonin-permeabilized cells is inhibited by synthetic peptides containing these NLSs (Kann et al., 1999). Binding of the capsid to the NPC was confirmed by blocking the NPC with WGA and antibodies against Nups, and was reconstituted by performing the nuclear import assay in the presence of recombinant importin-α and β (Kann et al., 1999). Since in the semipermeabilzed cells the HBV capsid binds to the NPC without migration into the nucleus, EM of *Xenopus* oocytes microinjected with phosphorylated recombinant capsids was used to visualize where at the NPC the capsid binds (Pante and Kann, 2002). It was shown that HBV capsids not only bind to the NPC, but are able to cross the NPC intact, without disassembly (Pante and Kann, 2002; **Figure 2**). However, although these recombinant capsids are able to cross the NPC, they are unable to be released into the nucleus, and instead get arrested within the NPC nuclear basket (**Figure 2**).

In addition to the experiments with recombinant HBV capsids, further experiments were performed with isolated HBV capsids from cells transfected with a plasmid containing the HBV genome (Rabe et al., 2003). HBV contains a DNA genome, but replicates by means of an RNA pregenome (Seeger et al., 2013). Thus during infection, HBV capsids in the cytoplasm of infected cells have their genome at different stages of maturation (immature capsids), but only those with complete genome maturation (mature capsids) form progeny viruses that are released from the infected cell (Gerelsaikhan et al., 1996). Rabe et al. (2003) purified immature capsids from the cytosol of HBV-transfected cells and extracellular mature capsids, and used these in digitonin-permeabilized cells in combination with fluorescence *in situ* hybridization (FISH) to detect the viral genome. It was found that mature capsids enter the nucleus and are able to undergo genome uncoating, DNA nuclear import, and replication (Rabe et al., 2003). Immature capsids on the other hand, were able to bind to the NE of semipermeabilized cells, but did not show release of the immature genome (Rabe et al., 2003). Repeating the digitonin-permeabilized assay with mature capsids in the presence of recombinant importin-α and β, and Ran, it was determined that genome uncoating from the mature capsid is Ran-independent (Rabe et al., 2003). Using the EM *Xenopus* nuclear import assay it was further demonstrated that, similar to the recombinant capsids, both the mature and immature capsids are translocated through the NPC into the nuclear basket; however, immature capsids remain arrested within the nuclear basket, while mature capsids are released into the nucleus (Rabe et al., 2003; **Figure 2**). These results reveal that HBV uses a well-coordinated strategy to deliver only mature viral DNA into the nucleus. Capsids containing immature viral genomes cross the NPC but are trapped at the NPC nuclear basket and are not released into the nucleus until the genomic double stranded DNA is completed. These results also show that HBV capsids are an exception from other nuclear import cargos that use the cellular transport receptors importin-α and β, in that the NPC translocation of HBV capsid is mediated by these importins but not by Ran.

To determine which components of the NPC are involved in HBV capsid retention at the NPC nuclear basket, and in capsid disassembly, Schmitz et al. (2010) performed immunoprecipitation assays with different type of capsids and rat liver nuclear extracts, and found that all type of capsids precipitated the nuclear basket protein Nup153 (Schmitz et al., 2010). Evidence of a direct interaction of the capsid with Nup153 was demonstrated by performing *in vitro* binding assays with purified capsids and recombinant Nup153, as well as with different fragments of Nup153 (Schmitz et al., 2010). Interestingly, binding of capsids is to the importin-β binding region of Nup153, implying that HBV capsids and importin-β compete for Nup153 binding (Schmitz et al., 2010). Indeed, competition experiments suggested that Nup153 is responsible for releasing importin-β from the capsids (Schmitz et al., 2010). Since all these were results from *in vitro* biochemical assays, the authors further determined the involvement of Nup153 in capsid arrest at the nuclear basket by performing experiments with cells in which Nup153 has been partially depleted by RNAi, and found that a significant portion of recombinant phosphorylated capsids were no longer retained in the nuclear basket, but instead entered the nucleus of these cells (Schmitz et al., 2010). Furthermore, cross-linked mature capsids do not interfere with capsid binding to the NPC but prevent their entry into the nucleus (Schmitz et al., 2010). Therefore, based on these results the authors proposed a mechanism for capsid disassembly in the nuclear basket whereby mature HBV capsid uses importin-α and β to cross the NPC, where, upon reaching the nuclear basket, dissociation of the importins from the capsid allows it to directly bind to Nup153, with some core proteins binding all available Nup135 sites, resulting in capsid disassembly and release of the uncoated DNA and the remaining core proteins into the nucleus (Schmitz et al., 2010).

In summary, HBV capsids use the cellular nuclear import machinery (NPC, and importin-α and β) to deliver their genome into the nucleus, but in contrast to cellular cargo, the capsid is not released into the nucleus but binds directly to Nup153 at the nuclear basket, and this somehow triggers disassembly of the capsid and nuclear entry of the viral genome (**Figure 1A**). The disassembly step is genome-dependent: while mature capsids disassemble, immature capsids remain arrested in the nuclear basket. However, it remains to be determined how Nup153 triggers capsid disassembly and preferentially disassembles mature capsids instead of immature capsids.

## Herpes Simplex Virus 1

Herpesviruses are a large family of viruses, of which HSV1 is the best characterized for its nuclear import strategy. HSV1 is an enveloped virus containing an icosahedral capsid (120 nm), a double-stranded DNA genome of 152 kbp, and a proteinacious layer (called the tegument) between the capsid and envelope (Roizman et al., 2013). HSV1 enters the host cell by either fusion of the viral envelope with the plasma membrane or endosomal membrane after endocytosis (reviewed by Campadelli-Fiume et al., 2012). The DNA-containing capsid and its surrounding tegument is then released into the cytoplasm, with some of the tegument proteins immediately dissociating from the capsid and others remaining more tightly bound to the capsid, and is transported along microtubules, using dynein, to the nuclear periphery (Sodeik et al., 1997; Dohner et al., 2002). The capsid with some of its tegument proteins then docks at the NPC, and this binding somehow triggers ejection of the genome from the capsid. The DNA is subsequently transported to the nucleus through the NPC and the capsid devoid of DNA (empty capsid) is left at the NPC cytoplasmic periphery and eventually is released into the cytoplasm (**Figure 1B**).

**FIGURE 3:**
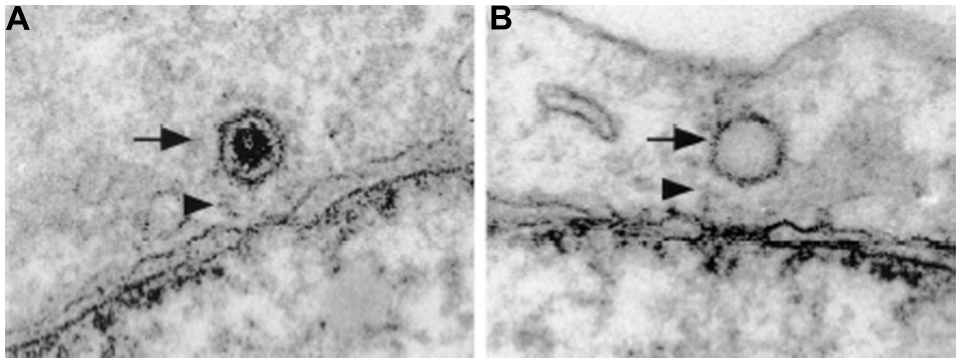
**Herpes simplex virus 1 capsids dock at the cytoplasmic side of the NPC.** Electron micrographs of NE cross-sections from Vero cells infected with HSV1. Both DNA-containing capsids **(A)** and empty capsids **(B)** are located in close proximity to the NPC. HSV1 capsids bind to the NPC with a distinct orientation with one of the vertices facing the NPC. Arrows point to HSV1 capsids and arrowheads point to NPC cytoplasmic filaments. Reproduced with permission from Ojala et al. (2000).

Several studies have shown that the HSV1 capsid binds to the NPC cytoplasmic filaments. These use HSV1 infected cells, *in vitro* binding assays with HSV1 capsids and isolated rat liver nuclei, and injection of HSV1 into *Xenopus* oocytes (Sodeik et al., 1997; Ojala et al., 2000; Shahin et al., 2006). Binding of purified HSV1 capsids to isolated nuclei is blocked by anti-Nups antibodies and by WGA, confirming the EM observations that the capsid docks to the NPC (Ojala et al., 2000). In cross-section EM pictures the capsid is depicted bound to NPCs at a distance of about 50 nm away from the center of the NPC and it has a distinct orientation with one of the vertices facing the NPC (**Figure 3A**; Sodeik et al., 1997; Ojala et al., 2000). It was thus speculated that the HSV1 capsid binds to the cytoplasmic filaments of the NPC. More recently, EM of cells infected with HSV1 clearly shows the binding of the capsid to the NPC cytoplasmic filament (Bauer et al., 2013). In order to determine the specific NPC binding partners, Copeland et al. (2009) performed experiments with cells either preloaded with an anti-Nup358 antibody or depleted of Nup358 by RNAi and found reduced capsid binding to the NPC of HSV1 infected cells (Copeland et al., 2009). Reduced capsid binding to the NPC was also reported in cells depleted of Nup214 by RNAi (Copeland et al., 2009; Pasdeloup et al., 2009), but an antibody against Nup214 did not inhibit this binding (Copeland et al., 2009). Thus, incoming HSV1 capsids dock at the NPC is via binding to Nup358 and possibly Nup214, which are components of the NPC cytoplasmic filaments.

Two studies have addressed the cellular factors and viral proteins involved in the binding of the HSV1 capsid to the NPC (Ojala et al., 2000; Copeland et al., 2009). To determine cellular factors, Ojala et al. (2000) first performed *in vitro* binding assays with purified capsids and isolated nuclei in the presence or absence of cytosol, in the presence of anti-importin-β antibodies, or in the presence or absence of recombinant importin-β, and determined that capsid binding to the NPC is importin-β dependent. Moreover, performing similar experiments but adding RanQ69L, a dominant-negative mutant of Ran unable to hydrolyze GTP, it was found that the docking of the capsid to the NPC is also Ran dependent (Ojala et al., 2000). The purified capsids used in these assay were associated with tegument proteins; therefore, to test whether teguments proteins were involved in the docking of the capsid to the NPC, Ojala et al. (2000) performed an *in vitro* binding assay with trypsin-treated capsids, which have the tegument proteins removed, and found reduced binding of the trypsin-treated capsids to the NPC (Ojala et al., 2000). More recently, Copeland et al. (2009) found that microinjection of antibodies against the tegument protein VP1/2 (UL36) into cells prior to HSV1 infection inhibits the binding of the HSV1 capsid to the NPC, and Pasdeloup et al. (2009) showed that the minor capsid protein UL25 immunoprecipitates Nup214. Taken together, these data suggest that HSV1 capsid docking at the NPC is importin-β- and Ran-mediated, and involves the tegument protein VP1/2 and the capsid protein UL25. However, it remains undetermined whether VP1/2 and UL25 binds to importin-β directly.

The next step in the nuclear transport mechanism for the HSV1 genome is the ejection of the DNA from the capsid. In contrast to the capsid docking at the NPC step, less is known about this step. Initial studies showed that empty capsids are associated with the NPC (**Figure 3B**), presumably after DNA release (Sodeik et al., 1997), and that DNA release from the capsid requires the presence of cytosol and energy (Ojala et al., 2000). Although the exact trigger for DNA release has yet to be determined, Nup214 has been hypothesized as the possible cue for this because Nup214 depletion by RNAi results in a delay of viral genome delivery into the nucleus (Pasdeloup et al., 2009). In terms of which viral proteins are required, the involvement of the tegument protein VP1/2 in DNA release has long been known since a HSV1 temperature-sensitive mutant with a single amino acid change on VP1/2 (Abaitua et al., 2011) binds to the NPC, but does not release its DNA at the non-permissive temperature (Batterson et al., 1983). Furthermore, proteolytic cleavage of VP1/2 is required for the release of the DNA into the nucleus (Jovasevic et al., 2008). The capsid protein UL25 is also involved in facilitating nuclear import of the HSV1 genome, as overexpression of UL25 impairs DNA nuclear entry by competing with the UL25 protein in the virus (Rode et al., 2011). Interestingly, an *in vitro* EM study of HSV1 DNA uncoating demonstrated that DNA release occurs through the capsid portal (Newcomb et al., 2001), a ring structure formed by 12 copies of the tegument protein UL6 located at one of the capsid vertices (Trus et al., 2004; Cardone et al., 2007). Although the current data does not provide a full picture for the DNA release step, the evidence suggests that the interactions between VP1/2 and Nup358 first bring the capsid and NPC closer together, leading to an interaction between UL25 and Nup214, which then ultimately leads to the ejection of the viral DNA through the capsid portal.

How the HSV1 DNA translocates through the NPC is also unclear. However, based on the similarities of the mechanism of viral genome packing between HSV1 and bacteriophages it has been hypothesized that translocation through the NPC does not depend on NTRs, but instead the DNA is expelled from the capsid by pressure, and this pressure is the force that propels the DNA through the NPC (reviewed by Liashkovich et al., 2011). In support of this hypothesis it has been shown that similar to bacteriophages, the DNA end that is packed last, is the first to be ejected, and immediately transcribed (Newcomb et al., 2009). The DNA has also been visualized by atomic force microscopy as a condensed rod-like structure coming out of the nuclear basket (Shahin et al., 2006), thus possibly the ejection force makes the DNA cross the NPC as a rod-like structure. Moreover, by osmotically suppressing DNA release from HSV1 capsids, Bauer et al. (2013) showed for the first time that capsids have high internal pressure. Thus, it is possible that the pressure-driven DNA ejection pushes the DNA through the NPC.

In summary, HSV1 uses a unique mechanism to deliver its genome into the nucleus, which involves capsid binding to the NPC, DNA release from the capsid through the capsid portal, and transport of the DNA through the NPC possible by the pressure of DNA ejection from the capsid (**Figure 1B**). Much is left to be determined in this mechanism, including which viral proteins interact with importin-β, the exact trigger for genome release, and the exact mechanism for DNA translocation through the NPC.

## Baculoviruses

Baculoviruses are rod-shaped (30–60 × 250–300 nm), enveloped viruses with circular double-stranded DNA genomes ranging in size from 80 to 180 kbp that infect insects and other arthropods (Rohrmann, 2013). Baculoviruses are unique compared to other virus families because they have two infectious forms: the occlusion-derived virion (ODV), comprising enveloped virions embedded within a crystalline matrix of protein, and the budded virus (BV), comprising a capsid containing the genome (referred to as the nucleocapsid) surrounded by a membrane envelope acquired by budding at the plasma membrane (Friesen, 2007; Rohrmann, 2013). ODVs are involved in virus transmission between insect larvae and the BV is the infection form responsible for cell-to-cell transmission within the insect and in tissue culture cells. Although both the ODV and BV forms contain nucleocapsids enclosed within envelopes of different origins, it is the nucleocapsid that eventually gets released into the cytoplasm, moves within the cytoplasm using actin-polymerization, and delivers the genome into the nucleus. Despite the popular use of baculoviruses as protein expression vectors in biomedical research and its potential to be developed as a biopesticide, the detailed molecular mechanism by which this virus delivers its genome into the nucleus remains to be established.

Baculoviruses are classified based on their DNA sequence into four genera: *Alpha-*, *Beta-*, *Gamma-*, *and Deltabaculovirus* (Jehle et al., 2006; Herniou et al., 2011). *Autographa californica* nucleopolyhedrovirus (AcMNPV), the archetype species of the *Alphabaculovirus* genus, is the most studied baculovirus and the most commonly used viral vector for protein expression. In tissue culture cells, budded virions of AcMNPV enter the cell by endocytosis, and the low pH of endosomes triggers a conformational change in the viral fusion protein GP64, resulting in fusion of the viral and endosomal membranes, releasing the nucleocapsid into the cytoplasm (Rohrmann, 2013). In the cytoplasm, the WASP-like protein VP78/83, located at one end of the nucleocapsid, induces polymerization of actin, which then looks like a comet tail that propels the baculovirus toward the cell nucleus (Goley et al., 2006; Ohkawa et al., 2010). An earlier EM study using tissue cultured insect cells infected with AcMNPV found intact nucleocapsids in the nucleus [(Carstens et al., 1979), reviewed by (Au et al., 2013)]. However, it was not clear whether the nucleocapsids entered the nucleus at mitosis during NE breakdown. EM of larvae infected with AcMNPV also revealed intact nucleocapsids in the nucleus and associated with the NPC (Granados and Lawler, 1981), but it was unclear whether these were newly synthesized nucleocapsids. These caveats were addressed in a study using non-dividing mammalian cells infected with AcMNPV, which revealed nucleocapsids both in the nucleus and at the cytoplasmic side of the NPC (van Loo et al., 2001). These studies indicate that the intact nucleocapsid of AcMNPV enters the nucleus through the NPC (**Figure 1C**).

**FIGURE 4:**
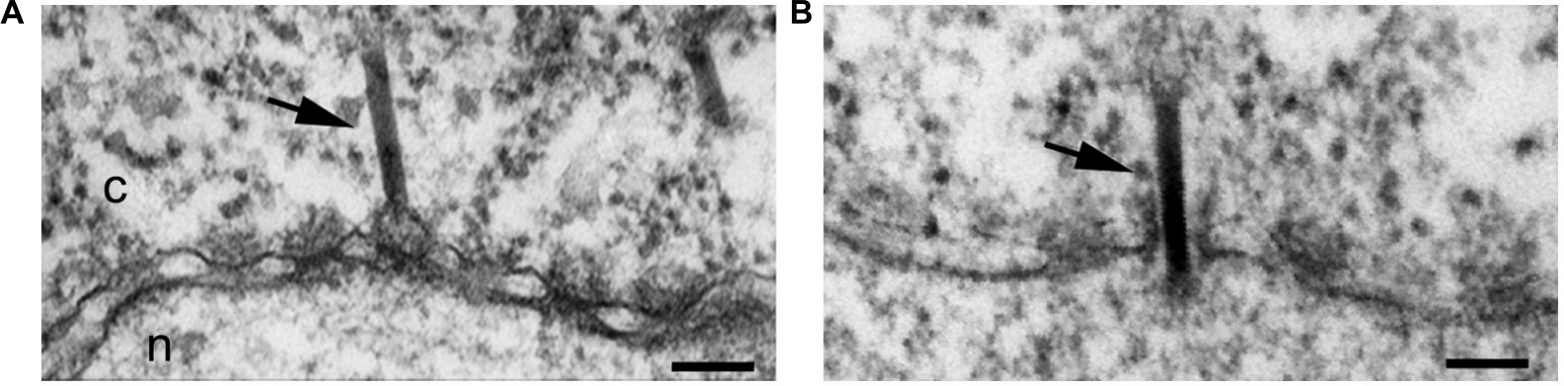
**Baculovirus AcMNPV capsids cross the NPC intact.** Electron micrographs of NE cross-sections from *Xenopus* oocytes microinjected with baculovirus AcMNPV capsids. Intact capsids (arrows) are detected docked at the NPC prior to nuclear import **(A)** and midway through the NPC **(B)**. Scale bars, 100 nm; n, nucleus; c, cytoplasm. Arrows point to capsids. Reproduced with permission from Au et al. (2013).

Two recent studies, one using fluorescently labeled AcMNPV and fluorescence microscopy (Ohkawa et al., 2010), and the second using the *Xenopus* oocyte nuclear import assay (Au and Pante, 2012), convincingly demonstrated that indeed the nuclear entry mechanism of the AcMNPV nucleocapsid involves the NPC. To determine that the NPC is the route of nuclear entry of the AcMNPV nucleocapsid, Ohkawa et al. (2010) used two experimental approaches: infection of cells pre-injected with WGA and infection of cells that expressed a dominant-negative mutant of importin-β, which binds to the NPC and blocks nuclear import (Gorlich et al., 1996). Both experiments yielded significantly less nucleocapsids in the nucleus than in the control untreated cells, but still few nucleocapsids were found in the nucleus (Ohkawa et al., 2010). Microinjection of *Xenopus* oocytes with purified AcMNPV nucleocapsids in combination with EM and electron tomography showed nucleocapsids vertically traversing the NPC and midway through the NPC (Au and Pante, 2012; **Figure 4**). At early time of injection the nucleocapsids were interacting with the NPC cytoplasmic filaments, and at late time of infection they were found in the nucleus. Furthermore, injecting WGA into the oocytes before injection of the nucleocapsid did not yield nucleocapsids in the nucleus, confirming that the only route for nuclear entry of the nucleocapsid is through the NPC (Au and Pante, 2012).

Although there is much experimental evidence demonstrating that intact AcMNPV nucleocapsids enter the nucleus through the NPC, early EM studies of *Betabaculovirus*-infected larvae revealed nucleocapsids docking at the NPC at different stages of releasing their genome, but not in the nucleus [(Summers, 1969, 1971), reviewed by Au et al., 2013]. This suggests a mechanism of DNA nuclear import similar to that used by HSV1, which attaches to the cytoplasmic side of the NPC and ejects its nucleic acid into the nucleus through the NPC, leaving empty capsids at the NPC.

In summary, viruses from the large *Baculoviridae* family can use different mechanisms for delivering their genome into the nucleus. Two mechanisms have been unveiled thus far. The first, used by *Alphabaculoviruses*, indicates that the intact nucleocapsid enters the nucleus through the NPC (**Figure 1C**). The second, used by *Betabaculoviruses*, involves docking of the nucleocapsid at the NPC, followed by ejection of the nucleic acid through the NPC leaving an intact empty capsid at the cytoplasmic side of the NPC. However, for both types of mechanisms further studies are required to determine the cellular and viral components involved.

## Nuclear Entry of Non-Enveloped DNA Viruses

**FIGURE 5:**
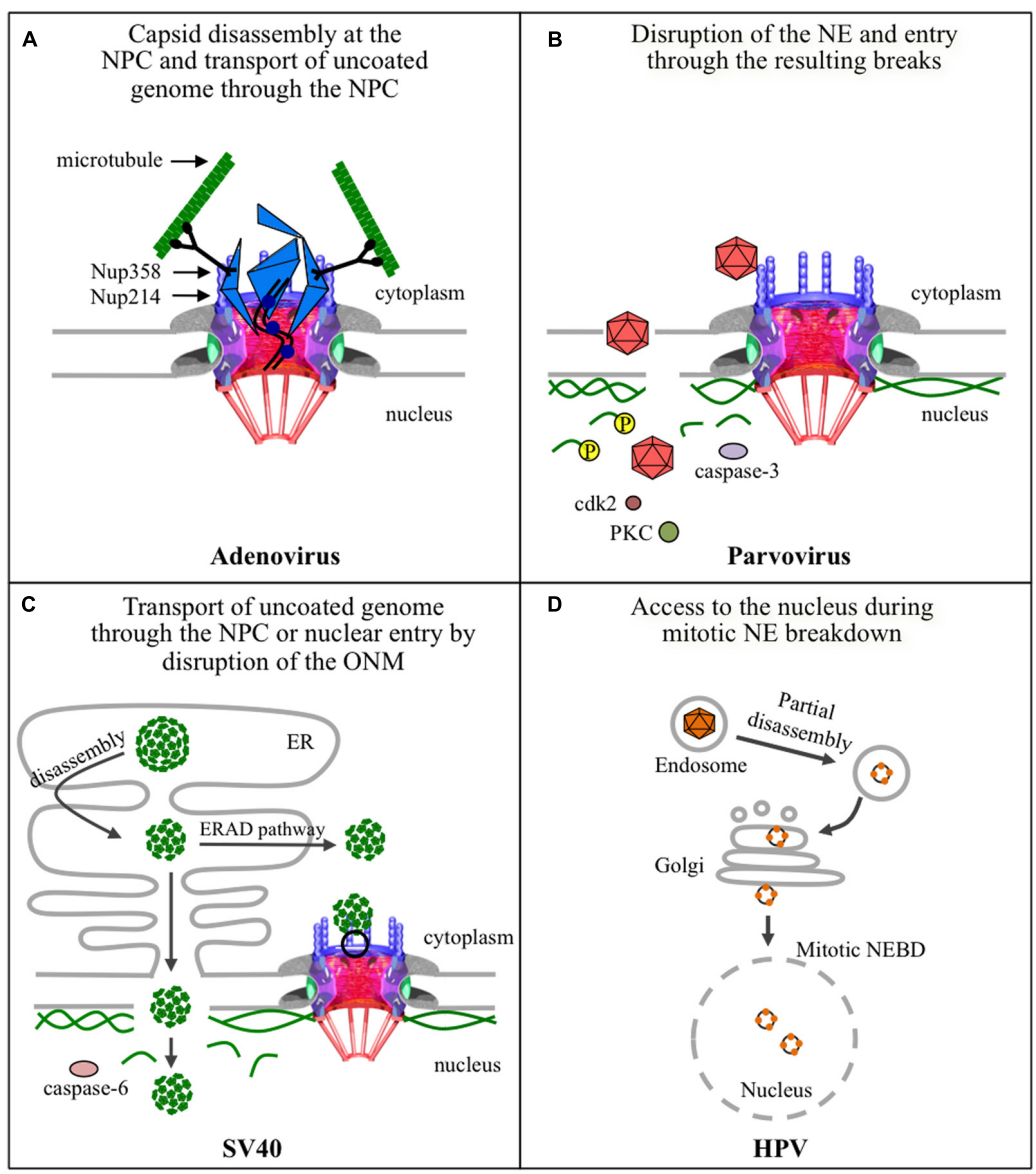
**Strategies used by non-enveloped viruses for nuclear entry of their genomes. (A)** The adenovirus capsid docks at the NPC via binding of hexon protein to Nup214, and uses Nup358-bound kinesin-1 via its heavy chain to completely disassemble the capsid that is also bound to kinesin-1, via its light chain, and deliver the uncoated genomes into the nucleus through the NPC. Kinase-1 binds to the capsid via its light chains and to Nup358 via its heavy chains. Movement of kinase-1 along microtubules exerts a pulling action that disassembles the capsid. **(B)** Parvoviruses enter the nucleus by transiently disrupting nuclear membranes, by a yet unknown mechanism, and disassembling the nuclear lamina. The latter involves phosphorylation of lamin A/C by PKC and cdk2, and cleavage of lamin-B by caspase-3. **(C)** SV40 partially disassembles inside the endoplasmic reticulum (ER) and the subviral particles could follow two different pathways to deliver the genome into the nucleus. The first involves exit of the subviral particle into the cytoplasm using the cellular ERAD pathway and cellular chaperones, disassembly at the NPC, and import of the uncoated genome through the NPC. The second is directly from the ER to the nucleus by direct disruption of the inner nuclear membrane and underlying nuclear lamina using caspase-6. **(D)** HPV partially disassembles in endosomes, and a possible route through the Golgi apparatus, and gains access to the nucleus during mitosis when the NE breaks down.

Non-enveloped viruses in general enter their host cells through an endocytic pathway and must escape from endosomes into the cytoplasm, or they risk degradation in the host lysosome. Viruses such as, adenoviruses and parvoviruses, have enzymes in their capsids that become exposed or active in the acidic environment of endosomes, ultimately leading to the disruption of endosomal membranes and the release of the virus into the cytoplasm. SV40 on the other hand, has a unique strategy for cytoplasmic release: instead of escaping from endosomes, it enters the ER, from which it travels to the cytoplasm or possibly to the nucleus. Interestingly, HPV either requires the acidic endosomes in order to escape to the cytoplasm or is routed to the Golgi in order to then travel to the nucleus. As summarized in **Figure 5**, although these four non-enveloped viruses may share some structural features or similar entry pathways, each virus uses a distinct mechanism for the delivery of its genome into the nucleus.

## Adenoviruses

**FIGURE 6:**
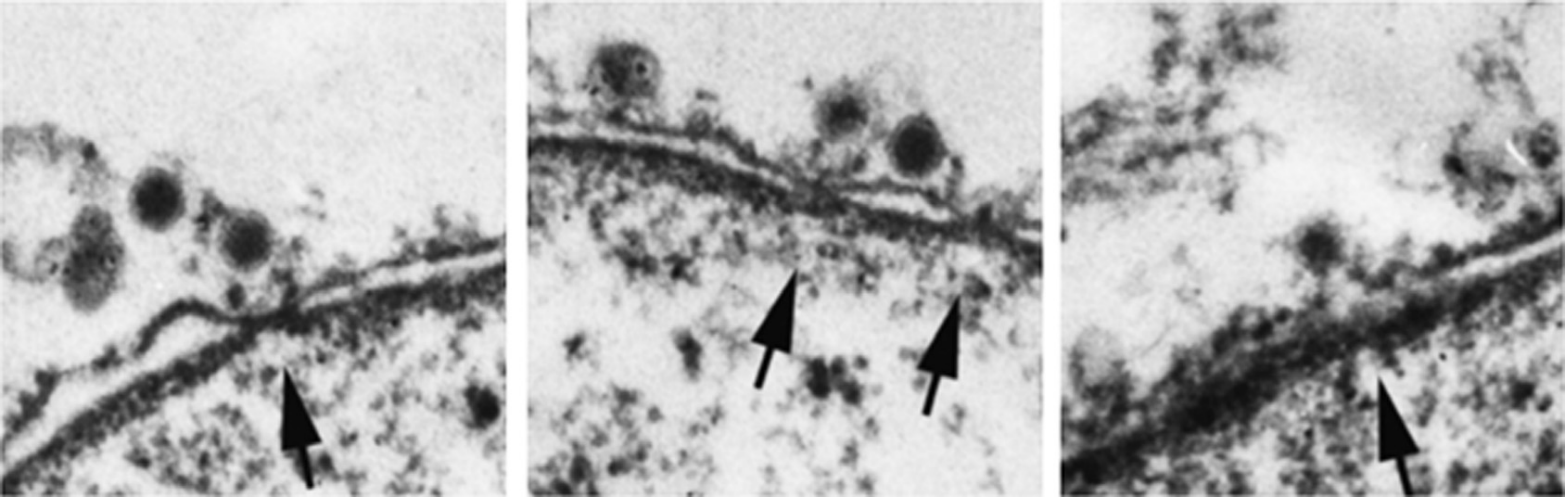
**Adenovirus capsids dock at the NPC cytoplasmic face before disassembly.** Electron micrographs of NE cross-sections from digitonin-permeabilized cells incubated with adenovirus capsids and cytosol. Arrows point to NPCs. Reproduced with permission from Saphire et al. (2000).

Adenoviruses are one of the largest (90–100 nm) and most complex non-enveloped viruses. They contain a double-stranded linear DNA genome of about 36 kbp that encodes more than 40 proteins, but only 13 of these are found in the virion (Benevento et al., 2014). The DNA is condensed by viral proteins, V, VII, and X, forming a nucleoprotein core that is packed into an icosahedral capsid of about 90 nm in diameter (Berk, 2013). The most abundant protein of the capsid is the hexon protein (protein II), with 720 copies in each virion (Benevento et al., 2014). Although there are over 57 human adenovirus serotypes, the most studied are serotype 2 and 5. One unique feature of adenovirus is the presence of fibers protruding from the 12 vertices of the capsid. For cell entry, the knobs at the end of these fibers attach to cellular receptors in the plasma membrane of host cells; this is followed by clathrin-mediated endocytosis of the virus and escape from endosomes by the lytic activity of protein VI found inside the capsid (reviewed by Leopold and Crystal, 2007; Smith et al., 2010). During cellular uptake the fibers dissociate from the capsid (Greber et al., 1993; Nakano et al., 2000), which disassembles further in the endosome and during endosomal escape (reviewed by Suomalainen and Greber, 2013). After being released into the cytoplasm, the partially disassembled capsids are transported along microtubules to the nuclear periphery where they dock at NPCs (reviewed by Leopold and Crystal, 2007; Smith et al., 2010). Capsid binding to the NPC (**Figure 6**) results in complete capsid disassembly and subsequent nuclear import of the viral genome (**Figure 5A**).

Docking of the partially disassembled adenovirus capsid at the NPC is via the binding of hexon protein to the NPC cytoplasmic filament protein Nup214. This was first demonstrated by Trotman et al. (2001), who used an *in vitro* binding assay incubating isolated nuclei or purified NE with adenovirus capsids or purified adenovirus proteins. In this assay neither cytosol nor importin-α/β are required for the binding of the adenovirus capsid to purified NE. To show that the NPC was involved in the *in vitro* binding of the capsids to the NE, experiments were performed in the presence of several anti-Nup antibodies, and it was found that antibodies against Nup214 block the binding of the adenovirus capsid to purified NE (Trotman et al., 2001). Furthermore, detection of hexon protein (to assess capsid disassembly) by immunofluorescence and viral DNA by FISH (to detect the viral genome) in cells microinjected with anti-Nup214 antibodies and infected with adenovirus showed that these antibodies prevent capsid disassembly and nuclear import of the adenoviral genome (Trotman et al., 2001). Binding of partially disassembled adenovirus capsid and purified hexon to Nup214 has recently been confirmed, using the nuclear import assay with digitonin-permeabilized cells depleted of Nup14 by RNAi (Cassany et al., 2015). These authors also studied adenovirus DNA nuclear import combining the digitonin-permeabilized assay with FISH, and found that Nup214, but not Nup358, was required for binding of adenovirus to the NE, for capsid disassembly, and for efficient nuclear entry of the adenovirus genome (Cassany et al., 2015). Moreover, these authors narrowed down the Nup214 binding site to a 137-amino-acid segment in the N-terminal domain of Nup214 by performing several experiments, including rescue experiments with the digitonin-permeabilized Nup214-depleted cells in the presence of several fragments of recombinant Nup214, adenovirus infection of cells overexpressing different fragments of Nup154, and *in vitro* binding assays with purified hexon and recombinant Nup214 fragments.

The disassembly of adenovirus capsids at the NPC has been shown to be mediated by kinesin-1 and to require the NPC cytoplasmic filament protein Nup358 (Strunze et al., 2011). Indirect immunofluorescence microscopy yields cytoplasmic co-localization of Nup358, Nup214, and Nup62 with disassembled virus particles 3 h post-infection (Strunze et al., 2011). Co-localization of Nups with disassembled capsids was also observed at the cell periphery of adenovirus-infected cells. To explain how the disassembled capsids, which are too large to diffuse through the cytoplasm, reach the cell periphery, Strunze et al. (2011) performed immunolocalization of the anterograde microtubule motor kinesin-1, and found that cytoplasmic disassembled capsids, detected with an antibody against disrupted but not intact capsids, indeed co-localize with kinesin-1 light chain in infected cells. Moreover, kinesin-1 light chain was also detected at the cytoplasmic periphery of the NPC together with capsids by immuonogold, and both overexpression of the C-terminus of kinesin-1 light chain, which is involved in binding to the transported cargo, and RNAi-mediated knockdown of kinesin-1 light chain reduce the amount of disassembled capsids found in the cytoplasm and adenovirus infection (Strunze et al., 2011). Further biochemical experiments demonstrated direct binding of the kinesin-1 light chain with adenovirus protein IX (Strunze et al., 2011). Since kinesin-1 heavy chain interacts with Nup358 (Cai et al., 2001), Strunze et al. (2011) studied adenovirus infection in both cells depleted of Nup358 by RNAi and cells overexpressing the kinesin-1 heavy-chain, which binds to Nup358 but not to the adenovirus capsid, and found that both conditions reduce adenovirus infection. Thus, it was proposed that disassembly of the adenovirus capsid at the NPC is mediated by the pulling action of kinesin-1, which is bound to Nup358 through its heavy chains and to the Nup214-docked capsid via its light chains (Strunze et al., 2011), when it moves along microtubules.

In addition to the displacement of Nups, Strunze et al. (2011) also found that the NPC permeability of adenovirus-infected cells increases; infection of cells that had been microinjected with large fluorescent dextrans, which are normally excluded from the nucleus, yielded nuclear localization of the dextran at 3 h post-infection (Strunze et al., 2011). Thus, they proposed that, in addition to disassembling the capsid, the movement of the Nup358- and capsid-bound kinesin-1 along microtubules also dissociates Nups from the NPC resulting in an increase of the NPC permeability (Strunze et al., 2011). The authors suggest that this increase in the NPC permeability may facilitate entry of the uncoated viral DNA into the nucleus (Strunze et al., 2011). However, several cellular factors and NTRs are involved in the nuclear import of the adenoviral genome, including hsp70, histone H1, importin-β, importin-7, and transportin-1 (Saphire et al., 2000; Trotman et al., 2001; Hindley et al., 2007). As these NTRs are known to bind to proteins and not to nucleic acids, and since adenoviral DNA is associated with several viral core proteins, the major core protein VII has been proposed as an adaptor between the viral genome and the NTRs (Wodrich et al., 2006). Indeed, transportin-1 mediates the nuclear import of recombinant mature protein VII in the digitonin-permeabilized cell nuclear import assay (Hindley et al., 2007). Thus, most likely the adenovirus genome enters the nucleus as a nucleoprotein using several NTFs, and the increased permeability of the NPC may speed this process.

In summary, adenovirus docks at the NPC by binding of hexon protein with the N-terminal domain of Nup214. Once docked at the NPC the capsid binds to the light chains of kinesin-1, which also binds Nup358 via its heavy chains. Movement of kinesin-1 along microtubules leads to capsid disassembly and NPC disruption through the pulling action of kinesin-1 (**Figure 5A**). Subsequently the uncoated viral genome associated with viral core proteins enters the nucleus through the NPCs that have increased permeability using several NTRs. More studies are needed to determine the actual mechanism of translocation of the viral DNA through the NPC.

## Parvoviruses

Parvoviruses are the smallest DNA animal virus, consisting of a single-stranded DNA genome of about 5 kb that is protected by a icosahedral capsid of 18–26 nm in diameter (reviewed by Berns and Parrish, 2013). The capsid is assembled from 60 copies of two (or three in some parvoviruses) size variants of the structural proteins VP1 and VP2, which are identical, except for the unique N-terminal sequence (140 amino acids) of VP1 (reviewed by Parrish, 2010). Parvoviruses in general use receptor-mediated endocytosis and escape from endocytic compartments into the cytoplasm by means of the enzymatic action of a phospholipase A2 (PLA2) motif in the unique region of VP1 (reviewed by Vihinen-Ranta and Parrish, 2006; Cotmore and Tattersall, 2007). Due to their small size, it was initially thought that parvoviruses enter the nucleus via the NPC. EM studies using the parvovirus minute virus of mice (MVM), however, have shown no evidence of entry through the NPC. Instead, a novel nuclear entry mechanism involving small disruptions of the NE was identified (**Figure 5B**; Cohen and Pante, 2005; Cohen et al., 2006).

**FIGURE 7:**
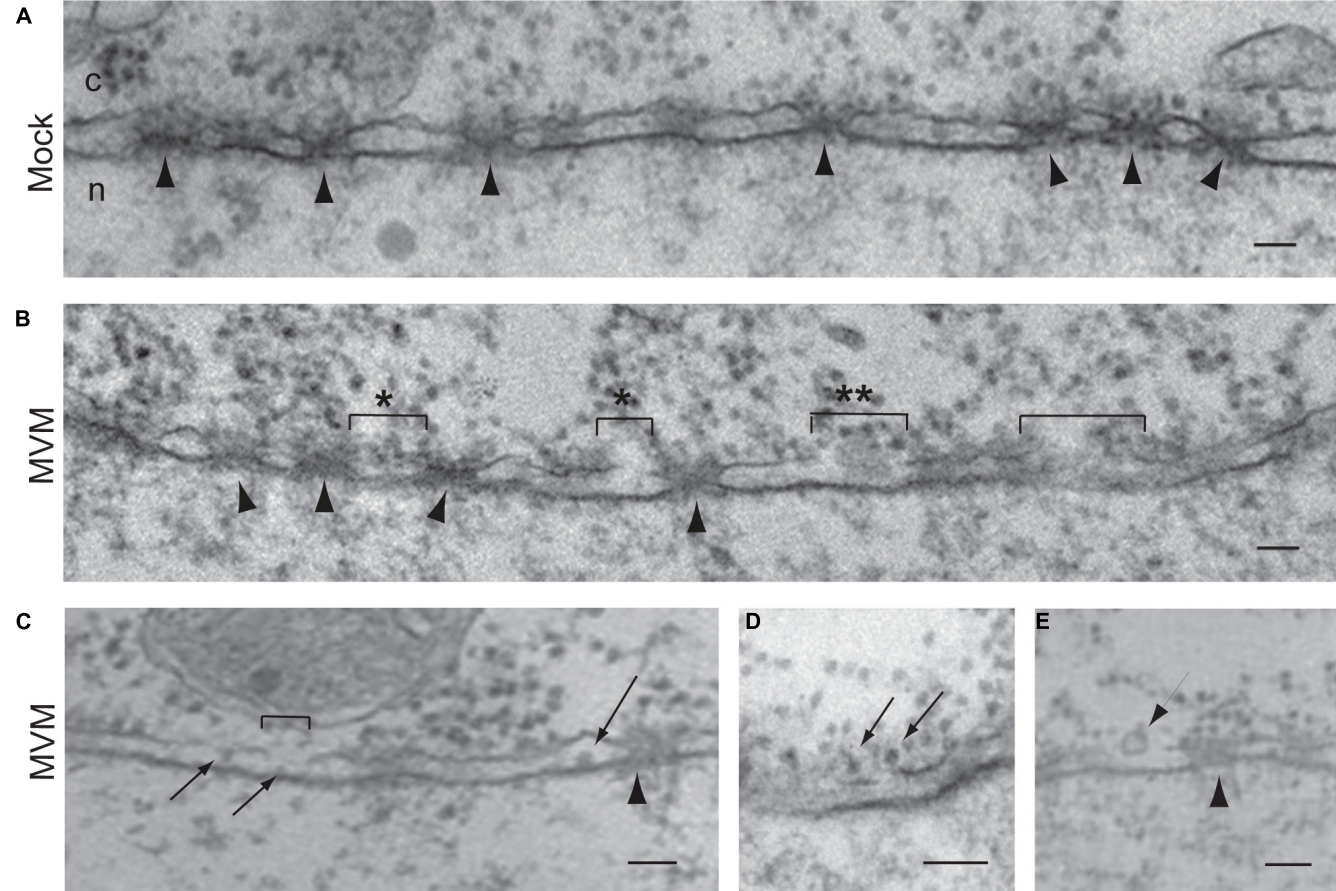
**Parvoviruses cause disruption of the NE for nuclear import.** Electron micrographs of NE cross-sections from *Xenopus* oocytes mock-injected **(A)** or injected with the parvovirus MVM **(B–E)**. Arrowheads point to NPCs. Brackets indicate breaks in the NE caused by MVM, which are often found close to NPCs (indicated by ^∗^). Virions are found close to the breaks (**B**, indicated by ^∗∗^), associated with the outer nuclear membrane (**D**, arrows), and in the perinuclear space (**C**, arrows). Small vesicles are found close to the breaks (**E**, arrow). Scale bars, 100 nm; n, nucleus; c, cytoplasm. Reproduced with permission from Cohen and Pante (2005).

Several studies using different parvoviruses and different experimental approaches have indicated that parvoviruses use an NPC-independent nuclear entry mechanism. A first study used incubation of purified nuclei with adeno-associated virus type 2 (AAV2) and found the virus in the nucleus even when the experiments were performed in the presence of WGA or antibodies against Nups, which both block the NPC (Hansen et al., 2001). Subsequent *Xenopus* oocyte import assays using MVM showed virions in the perinuclear space along with small (100–300 nm) disruptions of the NE (Cohen and Pante, 2005; Cohen et al., 2006; **Figure 7**). These NE disruptions were still seen even with WGA blocking the NPC, suggesting that disruptions in the NE are independent of the NPC (Cohen and Pante, 2005). Similar results were found by EM of MVM-infected cells, which also revealed virions in the perinuclear space and alterations in nuclear morphology (Cohen et al., 2006). Moreover, *Xenopus* oocyte import assays with other parvoviruses including canine parvovirus (CPV; Cohen et al., 2011a) and rat parvovirus H1 (Porwal et al., 2013) yielded similar results, indicating a possible conserved mechanism for nuclear entry among the parvovirus family. In addition to EM detection of NE disruptions, immunofluorescence microscopy and western blot analyses of MVM-infected cells show that the nuclear lamina immunostaining is disrupted (Cohen et al., 2006) and that lamin-B is cleaved (Cohen et al., 2011b). Interestingly, infected fibroblast cells show intact nuclear lamina by 21 h post-infection (Cohen et al., 2006, 2011b), indicating that the MVM-induced NE disruptions are transient. More recently, Porwal et al. (2013) developed a confocal microscopy method to quantifying chromatin release from the nuclei of digitonin-permeabilized cells that were incubated with several parvoviruses, including AAV2, CPV, and H1. This study confirmed that parvoviruses cause NE disruptions. Interestingly, AAV2 and H1 seem to differ in their mechanism for NE disruption, since for AAV2 but not H1, NE disruption was limited to virions that were exposed to pH 5.2 (Porwal et al., 2013).

More recently, scientist have tried to identify the mechanism by which parvovirus disrupt the NE. Since the only lipolytic enzyme of parvoviruses is the PLA2 activity found in the unique region of VP1, Cohen et al. (2011b) microinjected *Xenopus* oocytes with a mutated MVM for PLA2 activity (H42R, Farr et al., 2005) or with MVM treated with a drug that inhibits the PLA2 activity, and found NE disruptions in both experiments (Cohen et al., 2011b), indicating that the viral PLA2 is not responsible for MVM-induced NE disruption. Similar conclusions were drawn from the results of experiments performed by incubating digitonin-permeabilized cells with an AAV2 mutant with an inactivated PLA2 motif (Porwal et al., 2013). Since an enzyme of the virus did not cause the NE disruptions, attention was turned to cellular factors implicated in NE breakdown in cellular processes such as apoptosis and mitosis. Cohen et al. (2011b) adapted an *in vitro* NE breakdown assay (Muhlhausser and Kutay, 2007), which uses semipermeabilized cells expressing GFP-lamina-associated polypeptide 2β (GFP-LAP2β) as a NE marker, to screen for the effect of caspases inhibitors in MVM-induced NE disruption. In this assay, the permeability of the NE is measured as a nuclear influx of a fluorescently labeled 155-kDa dextran, which is too large to diffuse through the NPCs (Muhlhausser and Kutay, 2007). It was found that semipermeabilized cells showed a lack of dextran influx into the nucleus in the presence of caspase inhibitors and MVM, with the highest effect seen using a caspase-3 inhibitor (Cohen et al., 2011b). Subsequent studies co-injecting *Xenopus* oocytes with MVM and caspase inhibitors, and infecting cells with MVM in the presence of caspase inhibitor led to the conclusion that caspase-3 is involved in the unusual MVM nuclear entry mechanism (Cohen et al., 2011b). The role of caspase-3 is most likely in the proteolytic cleavage of lamin-B and not in the direct disruption of the nuclear membranes.

Similar studies using digitonin-permeabilized cells with their nucleus preloaded with a fluorescent 100 kD cargo, and measuring the nuclear fluorescence over time after incubation of the cells with the parvovirus H1 and inhibitors of mitotic enzymes involved in NE breakdown concluded that protein kinase C (PKC) and cyclin-dependent kinase 2 (cdk2) are involved in the mechanism of NE disruption induced by parvoviruses (Porwal et al., 2013). Furthermore, it was shown that PKC and cdk2 were activated with the addition of H1 (Porwal et al., 2013). Thus, it was proposed that the activated PKC phosphorylates lamin A/C, which activates cdk2 (further activated by caspase-3), leading to hyper phosphorylation of lamin A/C and disassembly of the nuclear lamina (Porwal et al., 2013). Thus, again the involvement of these enzymes explains the disassembly of the nuclear lamina that occurs during parvovirus infection, but not how the virus disrupts the nuclear membranes.

It has been proposed that although parvoviruses do not cross the NPC, they might interact with the NPC to cause NE disruption. This is based on indirect evidence for an interaction between Nups and parvoviruses: both H1 and AAV precipitate several Nups from a purified preparation of Nups (Porwal et al., 2013). Moreover, plugging the NPC with recombinant HBV capsids that attach to the nuclear basket without being released into the nucleus, inhibits H1-induced NE disruption (Porwal et al., 2013), arguing that the NPC is needed for parvovirus to cause NE ruptures. However, when the NPCs are blocked with WGA, H1-induced NE disruption was still present (Porwal et al., 2013). Thus, a direct interaction between parvovirus and the NPC during virus infection, and whether the NPC plays any role in the unusual mechanism of parvovirus nuclear entry, remain to be established.

A recent study using recombinant AAV2 (rAAV2) proposes that this virus enters the nucleus through the NPC (Nicolson and Samulski, 2014). This study found that blocking the NPC by microinjection of WGA into tissue culture cells partially inhibited the nuclear import of Cy5-labeled rAAV2 particles. This is in contrast to results with wild-type AAV2, for which WGA completely blocks the nuclear import of the virus (Hansen et al., 2001). The difference may be due to exposure of putative NLSs in recombinant but not wild-type virus. Several potential NLSs are located in the capsid proteins of AVV2 (Grieger et al., 2006) and other parvoviruses (Vihinen-Ranta et al., 1997; Lombardo et al., 2000; Pillet et al., 2003). However, since newly synthesized capsid proteins must reach the nucleus to permit assembly of new virus particles, these NLSs may be involved in the nuclear import of newly synthesized capsid proteins and not in the nuclear import of intact capsids. More studies are needed to explain why rAAV2 and wild-type AAV2 use different mechanisms for nuclear entry.

In summary, parvoviruses enter the nucleus by transiently disrupting nuclear membranes (**Figure 5B**), by a yet unknown mechanism that possibly involves Nups, followed by phosphorylation of lamin A/C by PKC and cdk2, and cleavage of lamin-B by caspase-3, ultimately causing disassembly of the nuclear lamina and nuclear entry of the virus. The potential contributions of Nups and the mechanism for NE disruption are two aspects that deserve more investigations.

## Simian Virus 40

Simian virus 40, the prototype of the *Polyomaviridae* family, is a small (45 nm in diameter) non-enveloped virus with a circular double-stranded DNA genome of about 5 kbp (DeCaprio et al., 2013). The genome is wrapped with cellular histone into a minichromosome, which is enclosed by the icosahedral capsid composed of 72 VP1 pentamers that are stabilized by interpentamic disulfide bonds (DeCaprio et al., 2013). Beneath the VP1 outer shell are the hydrophobic VP2 and VP3, which are not exposed on the virus surface. To enter the host cell, SV40 binds to gangliosides on the cell surface and is internalized in vesicles that then fuse with the ER delivering the virus to the ER lumen, where the capsid begins to disassemble using host disulfide isomerases (reviewed by Inoue and Tsai, 2013). From the ER, two nuclear entry pathways for SV40 have been described: one that involves the NPC after the virus escapes from the ER to the cytoplasm and another that is through direct disruption of the inner nuclear membrane from the ER lumen to the nucleus (**Figure 5C**).

**FIGURE 8:**
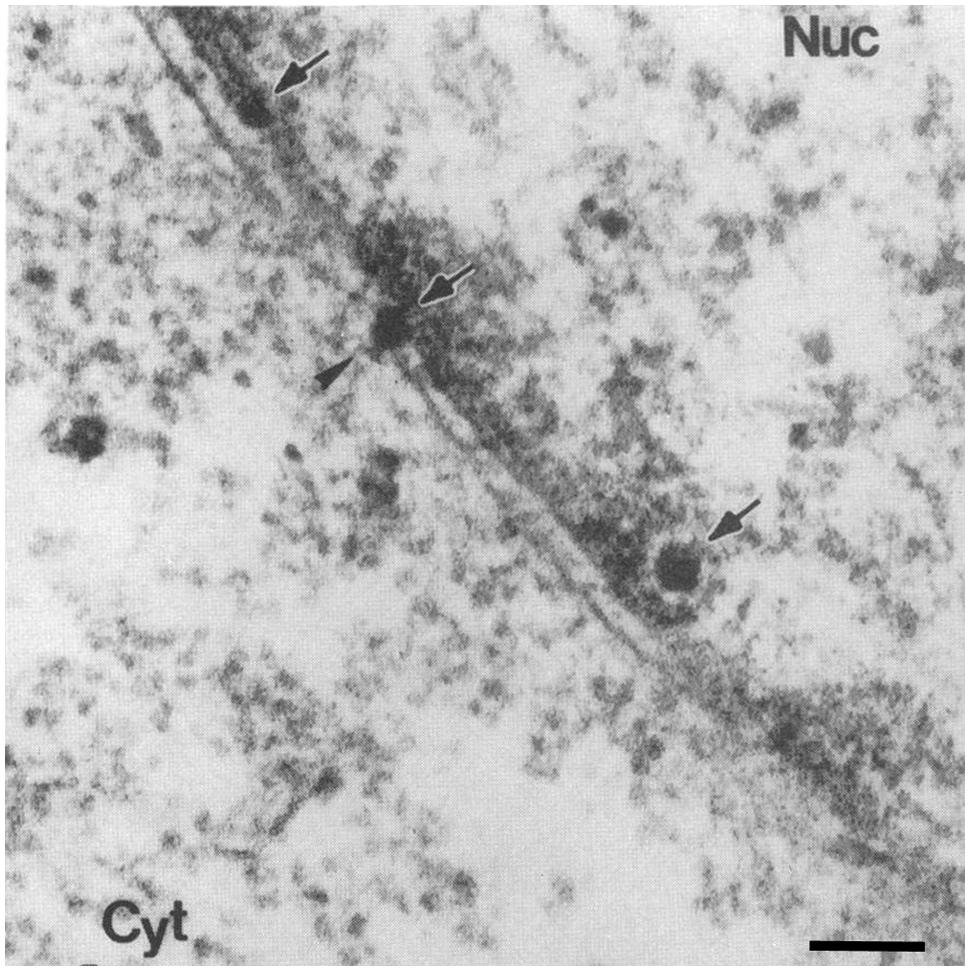
**Simian virus 40 is depicted at the NPC after microinjection.** Electron micrographs of a cross-section through the NE of a cell injected with SV40. Scale bars, 100 nm; Nuc, nucleus; Cyt, cytoplasm. Arrows point to virions, arrowhead points to a virion at the NPC. Reproduced with permission from Yamada and Kasamatsu (1993).

There are several lines of evidence that provide support for both nuclear entry pathways. An initial study, using cells microinjected with SV40, found that blocking the NPC by co-injection of WGA or antibodies against Nups prevents nuclear accumulation of VP1 and the viral large T-antigen (early gene product), suggesting that the nuclear entry process is NPC-dependent (Clever et al., 1991). Similar microinjection experiments combined with EM depicted virion at the NPC (Yamada and Kasamatsu, 1993; **Figure 8**). Because microinjection bypasses the normal entry route of the virion, it was not clear whether the virus crosses the NPC during an actual infection. Subsequently, it was demonstrated that when antibodies against the viral capsid protein VP2/3 are microinjected into the cytoplasm of SV40-infected cells there is a lack of nuclear accumulation of the viral T-antigen (Nakanishi et al., 1996), demonstrating that during an infection the virus enters the nucleus from the cytoplasm and suggesting that VP2/3 contains NLSs mediating the nuclear import of the virion. Indeed, VP3 contains an NLS that can bind to importin-α/β (Nakanishi et al., 2002). The role of this NLS in the nuclear import of the viral genome was tested in experiments using mutated virus-like particles that were formed from NLS defective VP3 in which all basic residues of the NLS were altered (Nakanishi et al., 2002). In these experiments cells infected with wild-type or VP3 mutated virus-like particles were subcellular fractionated at different hours post-infection, and the amount of full-length viral DNA accumulated in the cytoplasmic and nuclear fractions were measured. It was found that the DNA of the wild-type virus accumulates in the nucleus, whereas that of the VP3 mutated virus-like particles do not (Nakanishi et al., 2002). Similarly, virus particles that do not contain the coding region of VP2/3 do not transport their DNA into the nucleus (Nakanishi et al., 2007). Since VP2/3 is not found on the virus surface, it was important to determine whether VP2/3 is exposed after cell entry in order to then mediate nuclear entry. Co-immunoprecipitation experiments showed that the virion undergoes partial disassembly during viral entry, exposing the VP2/3 and allowing the VP3 NLS to then mediate nuclear entry of the viral DNA complexed to VP1-VP2/3 via cellular importins (Nakanishi et al., 2002). However, recent findings show that VP2/3 may not enter the nucleus along with the genome (Kuksin and Norkin, 2012). In this study, cells were infected with 5-Bromo-2-deoxyuridine- (BrdU) labeled DNA SV40, and the BrdU and VP2/3 were detected by indirect immunofluorescence microscopy (Kuksin and Norkin, 2012). Only SV40 DNA and not VP2/3 were found in the nucleus 12 h post-infection. Therefore, it was proposed that the viral DNA-protein complex might disassemble at the NPC before nuclear entry of the genome (Kuksin and Norkin, 2012).

Since evidence for the involvement of the NPC in nuclear entry of the viral genome requires that the virus be in the cytoplasm, several studies have elucidated the mechanism of viral escape from the ER to the cytoplasm. Virus disassembly first occurs within the ER using protein-disulfide isomerases and molecular chaperones, resulting in the exposure of VP2/3 (Schelhaas et al., 2007). The N-terminus of VP2 appear to be directly involved in the translocation of the virus across the ER membrane, since it is required for viral infection, creates pores within membranes, and therefore integrates into the ER membrane (Giorda et al., 2013). On the other hand, cellular proteins belonging to the ER-associated degradation (ERAD) pathway of misfolded proteins, BiP and BAP13, have also been implicated in translocation of the virus across the ER membrane (Geiger et al., 2011). This study showed that BiP and BAP13 are needed for VP2 mediated tethering of viral particles to the ER membrane (Geiger et al., 2011; reviewed by Byun et al., 2014). Furthermore, in support of this finding, other potential interaction partners were identified using immunoprecipitation, siRNA, and immunofluorescence microscopy techniques, including the cytosolic chaperone SGTA (small glutamine-rich tetratricopeptide repeat-containing protein α) and the two J-proteins, DnaJB14 and DnaJB12 (Walczak et al., 2014). Thus, there is adequate evidence for a model by which the partially disassembled virions traffic from the ER to the cytoplasm using the ERAD pathway.

The evidence suggesting direct entry of SV40 from the ER into the nucleus came from early EM studies showing NE disruptions near SV40 particles in the nucleus (Hummeler et al., 1970; Mackay and Consigli, 1976). Additional support for this model came recently with a study that found disruption to the nuclear lamina with SV40 infection (Butin-Israeli et al., 2011). In this study, immunofluorescence microscopy of non-dividing cells infected with SV40 shows gaps in the lamin-A/C immunostaining as early as 2 h post-infection (Butin-Israeli et al., 2011). Furthermore, fluctuations in the levels of lamin-A/C were also found by western blot within 10 h of SV40 infection. In order to determine the mechanism of nuclear lamina disruption, cells were infected with SV40 in the presence of several caspase inhibitors. It was found using immunofluorescence microscopy and western blot that a caspase-6 inhibitor prevents nuclear lamina disruptions and viral protein expression (Butin-Israeli et al., 2011). It had previously been found that caspase-6 is also activated during early infection (Butin-Israeli et al., 2010). Combining these findings confirms the model whereby SV40 leads to disruption of the nuclear lamina by activated caspase-6, ultimately leading to direct entry into the nucleus without leaving the ER. Thus, similar to parvoviruses, it appears that SV40 can also disrupt the nuclear lamina for nuclear entry of its viral genome; however, the molecular mechanism appears to be different. For example, SV40 infection involves lamin-A/C cleavage by caspase-6, whereas parvovirus infection involves lamin-B cleavage by caspase-3. Either way, similar to parvovirus infection, the molecular mechanism by which SV40 disrupts the nuclear membrane remains to be further elucidated.

In summary, the SV40 viral genome gains access to the nucleus through two potential pathways (**Figure 5C**). The first involves an NPC-dependent pathway, in which the partially disassembled virion exists the ER using the ERAD pathway, and through interaction between the VP3 NLS and importin-α/β is targeted to the NPC, completely disassembles at the NPC, and the uncoated viral DNA enters the nucleus through the NPC. The second pathway is through direct perforation of the inner nuclear membrane by partial disassembled subviral particles in the ER, followed by cleavage of lamin-A/C by caspase-6 and disruption of the nuclear lamina. Both pathways are possible and deserve further investigation to elucidate their molecular mechanisms.

## Human PapillomaVirus

Papillomaviruses are a large family of viruses of which the majority are HPVs (Bernard et al., 2010). The 55-nm icosahedral capsid is formed by 360 copies of the major structural protein L1 that assembles into pentamers, and variable amounts of the minor structural protein L2 (Howley et al., 2013). The genome is circular, double-stranded DNA of ∼8 kbp. In general, studies on early steps of HPV infection, especially the mechanism for nuclear entry, have been challenging and limited (reviewed by Day and Schelhaas, 2014). This is in part because viral production in cell culture systems yields limited amount of virions, due to the restriction of HPV productive life cycle to terminally differentiating keratinocytes (reviewed by Day and Schelhaas, 2014). Therefore, many studies use surrogate viral particles, either non-infectious virus-like particles (Kirnbauer et al., 1993) or pseudovirions that contain a plasmid encoding a reporter gene (Buck and Thompson, 2007; reviewed by Day and Schelhaas, 2014). Nevertheless, it has been established that L1 mediates the binding of the virus to the host receptor heparan sulfate proteoglycans, which initiates conformational changes in L1 and exposes L2 leading to internalization of the virus using multiple cellular pathways, including clathrin- and non-clathrin mediated endocytosis (reviewed by Sapp and Bienkowska-Haba, 2009; Day and Schelhaas, 2014). HPV virions then partially disassemble in the acidic environment of endosomes, leading to separation of L1 and L2, and the subviral particle consisting of L2 and the genome is then either routed to the Golgi apparatus or L2 disrupts the endosomal membrane and the subviral particle is released in the cytoplasm (reviewed by Sapp and Bienkowska-Haba, 2009; Day and Schelhaas, 2014).

Up until recently, very little was known about the nuclear entry of HPV. Several studies have documented that L2 from HPV11 and HPV16 contains two NLSs that mediate nuclear import when fused with GFP and interact with NTFs (Darshan et al., 2004; Bordeaux et al., 2006; Mamoor et al., 2012). In addition, L2 and the viral genome co-localize in the nucleus of cells infected with HPV, suggesting that they enter the nucleus as a complex (Day et al., 2004). However, no direct evidence for the use of the L2 NLSs during nuclear import of the viral genome has been shown. Instead, a second scenario has been proposed that involves NE breakdown during mitosis for the virus to access nuclear components required for viral replication (**Figure 5D**). The initial evidence for this mechanism came from a screen of a large library of bioactive compounds, which found that cell cycle inhibitors completely blocked HPV infection (Pyeon et al., 2009). Subsequently, it was showed that cell cycle arrest by serum starvation or using drugs inhibits HPV infection (Pyeon et al., 2009). More specifically, early prophase rather than late prophase or metaphase was found to be important for HPV infection (Pyeon et al., 2009). A CDK1 inhibitor was also found to block HPV infection in a dose-dependent manner, suggesting that phosphorylation of NE components by CDK1 and NE breakdown during early prophase is important for HPV nuclear entry and ultimately infection (Pyeon et al., 2009).

Further evidence for the dependence of HPV infection on NE breakdown during mitosis came from a recent high-throughput RNAi screen that found host mitotic and cell cycle regulator factors involved in HPV infection (Aydin et al., 2014). In this study, the requirement for mitosis during HPV infection was further tested, by infecting interphase cells with HPV16 pseudovirus containing pseudogenomes encoding GFP and monitoring early steps of infection by immunofluorescence microscopy. In interphase cells, the virus was endocytosed, moved to the perinuclear area, and partially uncoated, but only when mitosis was initiated would the viral DNA enter the nucleus (Aydin et al., 2014). More importantly, co-infection of S-phase-arrested cells with HPV and the parvovirus H1, which induces NE disruption, yield a detectable number of cells infected with HPV, suggesting that the NE disruption by H1 is sufficient for nuclear entry of HPV DNA (Aydin et al., 2014). Thus, the authors proposed that NE breakdown during mitosis is the step required for nuclear entry of HPV. Furthermore, because L2-GFP associate with host cell condensed chromatin from metaphase plate formation through cytokinesis, it was proposed that the subviral complex waits in the perinuclear area until NE breakdown occurs in order to enter the nucleus and gain access to nuclear components (Aydin et al., 2014).

In summary, NE breakdown during mitosis might facilitate the nuclear entry of HPV (**Figure 5D**). Unlike parvoviruses, however, HPV does not induce NE disruption, but rather must wait for the cell to disassemble the NE during mitosis in order to access nuclear components and replicate its genome. This strategy for nuclear entry is also used by retroviruses, such as the murine leukemia virus (reviewed by Cohen et al., 2011a).

## Concluding Remarks

Even though all known DNA viruses use very similar mechanisms to enter their host cells, they have evolved unique mechanisms to deliver their genome into the nucleus of their host cells. Recent studies on nuclear import of viruses have drastically changed the perception of how viruses use the cellular machinery for nuclear import. What was once thought to be just two or three common mechanisms for several viruses has now revealed to include a completely different and unique strategy for each virus to deliver its genome into the nucleus. This is in part due to the use of more advanced and correlative techniques, the emergence of alternative cell lines, and the development of more consistent wild-type and mutant virus production protocols between research groups. It is intriguing that not only the strategies between virus families are different, but that some viruses within the same family, as is the case for baculoviruses, may even use different strategies. It is fascinating that even some individual viruses, such as SV40, may use several different nuclear import strategies.

From comparison of some of the best-characterized viral nuclear import pathways, it is also evident that although some viruses use a similar strategy, the cellular components used could be different. For example, parvoviruses and SV40 both disrupt the nuclear lamina to deliver their genomes into the nucleus, however, parvoviruses use both phosphorylation and proteolytic cleavage of nuclear lamins, which require PKC, cdk2, and caspase-3, whereas SV40 uses proteolytic cleavage of lamin-A/C by caspase-6. It is also becoming evident that some viruses use the NPC and binding to Nups as a cue for disassembly. This is the case of the HBV capsid that disassembles at the nuclear basket after binding to Nup153, or HSV1 that ejects its genome by a possible binding of the capsid to Nup214. Although the picture for nuclear entry mechanisms is now clearer for many viruses, much remains to be explored. For example, the cellular factors and viral proteins involved in baculovirus nuclear entry, the factors that triggers HSV1 genome release from the capsid, how the different viral DNA translocate across the NPC for viruses that uncoat their genome before nuclear entry, and how parvoviruses and SV40 initially disrupt the NE, are among the many aspects of nuclear entry that remain undetermined.

Viruses are excellent models to understand cellular processes. The study of nuclear import of viruses could lead to new insights into the detailed mechanism by which molecules, including endogenous DNA, translocate through the NPC central channel, which is still unclear and a much-debated topic in the field of nuclear transport. Moreover, a detailed characterization of the nuclear import of viruses is an important step in the development of antiviral therapy that may successfully resolve viral diseases by interrupting entry of the viral genome into the nucleus of infected cells. Additionally, since viral vectors – containing a gene of interest – must also enter the cell’s nucleus to allow for gene expression, studies of nuclear import of viral genomes could help in the design of more efficient vectors for gene therapy. Thus, by learning about viruses and how they target their genome into the cell nucleus, we can learn about cell biology mechanisms, find antiviral targets for some viruses, and improve therapeutic potential for others.

## Conflict of Interest Statement

The authors declare that the research was conducted in the absence of any commercial or financial relationships that could be construed as a potential conflict of interest.
